# Sensitization of Resistant Cells with a BET Bromodomain Inhibitor in a Cell Culture Model of Deep Intrinsic Resistance in Breast Cancer

**DOI:** 10.3390/cancers15072036

**Published:** 2023-03-29

**Authors:** Balraj Singh, Vanessa N. Sarli, Ryan D. Milligan, Hannah E. Kinne, Anna Shamsnia, Laura J. Washburn, Sridevi Addanki, Anthony Lucci

**Affiliations:** 1 Department of Breast Surgical Oncology, The University of Texas MD Anderson Cancer Center, Houston, TX 77030, USA; 2 Morgan Welch Inflammatory Breast Cancer Research Program and Clinic, The University of Texas MD Anderson Cancer Center, Houston, TX 77030, USA

**Keywords:** inflammatory breast cancer, minimal residual disease, cancer relapse, cancer epigenome, metastasis prevention, intrinsic resistance, cell culture model, cancer quiescence, PD-L1, JQ1

## Abstract

**Simple Summary:**

Cell culture models of cancer typically favor proliferative but therapy-sensitive cells because body-like selection pressures are absent. To address this limitation, we previously described a function-based selection strategy to model deep intrinsic resistance in cultures of triple-negative breast cancer cells. Our methods were designed to reveal therapy-resistant, adaptable cancer cells that opportunistically switch between quiescence and proliferation. To determine the validity of this approach in identifying noncytotoxic drugs that could inhibit highly resistant breast cancer cells, we used our novel cell culture model to evaluate a well-known BET bromodomain inhibitor, JQ1, which modulates the cancer epigenome. JQ1 has been found to inhibit resistant cancer cells in several cancer types, including breast cancer. Low-dose JQ1 inhibited the growth of highly adaptable/resistant breast cancer cells in our cell culture model. Our results support the validity of a cell culture-based approach for modeling cancer.

**Abstract:**

We treated highly metabolically adaptable (SUM149-MA) triple-negative inflammatory breast cancer cells and their control parental SUM149-Luc cell line with JQ1 for long periods to determine its efficacy at inhibiting therapy-resistant cells. After 20 days of treatment with 1–2 µM of JQ1, which killed majority of cells in the parental cell line, a large number of SUM149-MA cells survived, consistent with their pan-resistant nature. Interestingly, though, the JQ1 treatment sensitized resistant cancer cells in both the SUM149-MA and SUM149-Luc cell lines to subsequent treatment with doxorubicin and paclitaxel. To measure JQ1-mediated sensitization of resistant cancer cells, we first eradicated approximately 99% of relatively chemotherapy-sensitive cancer cells in culture dishes by long treatments with doxorubicin or paclitaxel, and then analyzed the remaining resistant cells for survival and growth into colonies. In addition, combination, rather than sequential, treatment with JQ1 and doxorubicin was also effective in overcoming resistance. Notably, Western blotting showed that JQ1-treated cancer cells had significantly lower levels of PD-L1 protein than did untreated cells, indicating that JQ1 treatment may reduce tumor-mediated immune suppression and improve the response to immunotherapy targeting PD-L1. Finally, JQ1 treatment with a low 62.5 nM dose sensitized another resistant cell line, FC-IBC02-MA, to treatment with doxorubicin and paclitaxel.

## 1. Introduction

Many breast cancers, such as inflammatory breast cancer (IBC) and triple-negative breast cancer (TNBC), do not adequately respond to currently offered therapies. TNBC lacks the targeted therapy options available for hormone receptor-positive and HER2-positive breast cancers, so treatment relies on cytotoxic chemotherapy. A promising development for TNBC (and possibly for IBC) is the availability of immune checkpoint therapies such as those targeting PD-L1, although the response rate with such therapies is low and the duration of response is short [1]. Breast cancer patients who do not achieve a pathological complete response to neoadjuvant therapy carry minimal residual disease (MRD), which is often treatment-resistant, and are more likely to relapse [2,3,4]. To improve patient outcomes, we are investigating strategies that could decrease the probability of disease recurrence.

A major cause of therapy resistance, leading to an early relapse in triple-negative inflammatory breast cancer (TN-IBC), is deep intrinsic resistance in cancer cells. To meet the goal of advancing noncytotoxic broadly active therapeutic agents to overcome deep intrinsic resistance in a timely manner (prior to relapse), we have developed a cell culture-based approach for modeling deep intrinsic resistance. In this regard, with cancer progression being an evolution-like process [5,6,7,8,9], we select highly adaptable rare cells (0.01% subpopulation in a TN-IBC cell line) that are fit to survive in the body [10,11]. In the absence of glutamine, 0.01% of cells survive in quiescence for several weeks and then proliferate indefinitely. In support of the validity of this cell culture-based approach for modeling deep intrinsic resistance, we have found that IBC-derived TNBC cell lines (SUM149 and FC-IBC02) are a better source of highly adaptable cancer cells than non-IBC-derived TNBC cell lines (i.e., MDA-231 and its metastatic variants cultured from bone metastases in nude mice). This is evident from our observations that adaptable cells derived from IBC cell lines proliferate indefinitely in a glutamine-deficient medium, while adaptable cells derived from non-IBC cell lines fail to do so [10,11]. This result in cell culture mirrors the higher adaptability (and therapy resistance) of IBC than non-IBC in vivo.

We refer to the adaptable cancer cells selected in this manner as metabolically adaptable (MA) cells. SUM149-MA cells can also survive other metabolic challenges, such as a lack of glucose or oxygen, better than the parental cell line [10,11]. They are resistant to the chemotherapeutic drugs paclitaxel and doxorubicin and to several targeted therapies that inhibit cell proliferation [12]. Importantly, SUM149-MA cells are highly tumorigenic in nude mice, efficiently metastasizing to multiple organs such as the skin, lungs, and brain [11]. Molecular characterization has shown that they have activation of pathways that promote epithelial-to-mesenchymal transition (EMT) (e.g., high ZEB1, high SNAIL1, low GRHL2) and numerous changes affecting the epigenome (e.g., low TET2), alternative RNA splicing (e.g., low ESRP1, ESRP2), and RNA base modifications (e.g., high FTO) [12,13,14,15]. These characteristics suggest that body-like selection pressures select highly adaptable cancer cells that may drive therapy resistance. In the context of our cell culture model, genetic changes present in MA cells may cooperate with alterations in the epigenome, transcriptome, and proteome to provide different selection advantages over time and to confer deep intrinsic resistance.

Although SUM149-MA cells exhibit some useful features of poor-prognosis MRD, the problems inherent to cell culture still exist. The selection pressures imposed by the body occur one after another, a sequence that is not feasible to emulate in cell culture. Once a given challenge has been overcome in cell culture, proliferative cells, irrespective of their inherent adaptability, will have an advantage. To address this issue at a practical level, we rely on another evolution-related concept that is well-accepted in cancer research: the inverse correlation between cell proliferation and cell adaptability in biological systems. This concept also fits well with the cancer stem cell concept, wherein a small subpopulation of progenitor-like cancer cells (residing mostly in quiescence) drives the disease. Therefore, when we assay the efficacy of therapeutic drugs in cell culture, we assume that highly proliferative cancer cells are the first to be eliminated. Thus, by eliminating the vast majority of less-resistant cancer cells, we can reveal the resistant cells, which survive by switching to quiescence (a measurable phenotype and a characteristic of poor-prognosis MRD).

To provide a proof of concept for the usefulness of our cell culture model for evaluating cancer therapies, in the present study we evaluated a well-known BET bromodomain inhibitor, JQ1, which has been shown to favorably modulate the cancer epigenome to overcome therapy resistance in many leukemias and solid cancers [16,17,18,19,20,21]. Preclinical studies suggest that JQ1 may overcome resistance to other therapies in estrogen receptor-positive and triple-negative breast cancers [22,23]. The differentiation-state plasticity that often drives therapy resistance can be targeted by JQ1, which prevents changes to open chromatin architecture in basal-like breast cancer [24]. Briefly, the BET family proteins (BRD2, BRD3, BRD4, and BRDT) bind to chromatin via an interaction between their bromodomain motifs and acetylated lysine residues on histone tails and direct the assembly of macromolecular complexes involved in DNA replication, DNA damage repair, chromatin remodeling, and transcription. JQ1, a thienotriazolodiazepine, competitively binds to acetyl-lysine recognition motifs (bromodomains) on BET proteins, thus displacing them from chromatin [18]. Interestingly, JQ1 treatment may also enhance antitumor immunity, which involves changes in the expression of PD-L1 and PD-1 in tumor cells and immune cells [25,26]. Thus, the purpose of this study was to use our novel cell culture model of resistant MRD to assess the efficacy of JQ1 against metabolically adaptable TN-IBC cells and its immune-modulatory effects in these cells via PD-L1.

## 2. Materials and Methods

### 2.1. Cell Lines and Drugs

The resistant TN-IBC cell line used in this study was SUM149-MA, which was derived from a firefly luciferase-transfected SUM149 cell line (SUM149-Luc) [27]. We obtained the SUM149 cell line as a gift from Stephen Ethier (then at Barbara Ann Karmanos Cancer Institute, Detroit, MI, USA). The generation and characterization of these cell lines and their culture conditions were previously described [11,27]. We chose to use a luciferase-transfected cell line because it allowed us to image tumor growth and metastases from xenografts in nude mice in previous studies [11,27]. The resistant TN-IBC cell line FC-IBC02-MA, which was derived from the FC-IBC02 cell line [28], was also used. The generation and characterization of the FC-IBC02-MA cell line were previously described [10,14]. Since the initial selection of MA cell lines was performed using a glutamine-deficient medium, dialyzed fetal bovine serum (FBS) was used instead of regular FBS to further reduce the glutamine levels in the culture medium. Therefore, for consistency, all experiments for the present study were performed using media containing dialyzed FBS, even when the medium was supplemented with glutamine (as was carried out in parental cell lines and MA cell lines after the initial selection in the absence of glutamine).

We purchased JQ1 from APExBIO (Houston, TX, USA) and paclitaxel and doxorubicin from Sigma-Aldrich (St. Louis, MO, USA). We dissolved JQ1, paclitaxel, and doxorubicin in dimethyl sulfoxide (DMSO). We added equal volumes of DMSO without drugs to the cultures of the control cells. The DMSO volume was equal to or less than 0.04% of the volume of the culture medium.

### 2.2. JQ1 Treatment of Resistant Cancer Cells

SUM149-MA and SUM149-Luc cells were treated with JQ1 at 1–2 µM for 20 days. The effects of these treatments on cell growth and morphology were frequently monitored under a microscope. We observed substantially higher growth inhibition and cell killing in the parental SUM149-Luc cell line than in resistant SUM149-MA cells. Owing to the abnormal morphologies of cells after this treatment, it was difficult to clearly distinguish the dead cells from the live cells. Therefore, to obtain a better idea about the survival and growth ability of cells remaining after the treatment, we switched the JQ1-treated cell cultures to a regular drug-free medium and allowed the cells to recover and grow for 5–6 days. At the end of treatment, the cultured cells were stained with crystal violet and photographed or scanned.

### 2.3. Assays of Relative Resistance of Cells to Paclitaxel and Doxorubicin

To determine whether treatment with JQ1 affected the sensitivity of the resistant SUM149-MA and SUM149-Luc cells to paclitaxel and doxorubicin, we treated SUM149-MA and SUM149-Luc cells with 1 µM of JQ1 or DMSO for 6 days, allowed the cells to recover for 9 days, and then passaged them. The cells were then treated for 5–6 days with predetermined concentrations of paclitaxel (5 nM) or doxorubicin (25 nM), which killed 99% of the proliferating cells (confirmed by microscopic examination). We then removed the chemotherapeutic drugs by changing the medium and let the surviving cells form colonies for 12–13 days. We stained the colonies with crystal violet and counted them. In addition to the sequential treatments with JQ1 followed by chemotherapeutic drugs, we also analyzed the effect of a combination therapy involving JQ1 plus doxorubicin compared to doxorubicin alone.

### 2.4. Western Blotting

We performed Western blotting to detect protein bands as enhanced chemiluminescence signals on X-ray films, as previously described [29]. We used anti-PD-L1 (catalog number 13684; Cell Signaling Technology, Danvers, MA, USA) and anti-HSP90 (catalog number 4875; Cell Signaling Technology) antibodies for protein detection. After the detection of PD-L1, the Western blot membranes were re-probed to detect HSP90, which served as an internal control for the normalization of protein loading. Each Western blot was performed at least twice.

## 3. Results

### 3.1. JQ1 Resistance of SUM149-MA Cells

Evaluation of any drug in our cell culture model began by determining the appropriate dose range. Since epigenetic modulators are typically not cytotoxic drugs and require time to influence the phenotype, in pilot experiments we evaluated a broad range of doses to identify a dose that was noncytotoxic but influenced the phenotype (cell morphology, cell growth, cell death) over time, as observed by monitoring under a microscope. To compare the resistance to JQ1 of the SUM149-MA cells with that of parental SUM149-Luc cells, we treated cultures of both cell lines with different concentrations of JQ1 and monitored them under a microscope. We changed the drug medium as needed to remove floating dead cells from the culture dishes. Typically, when most of the cells were killed and about 1% of the cells were still attached to the dishes in the parental cell line, we shifted the cells to fresh medium without JQ1 and let the resistant cells recover and grow for a few days so that we could evaluate the relative resistance of these cells to JQ1 in both cultures.

**Figure 1:**
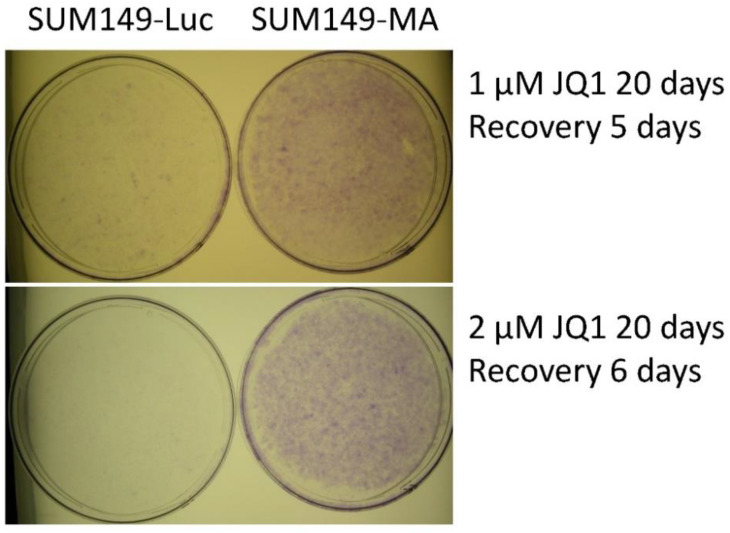
Resistance of SUM149-MA cells to treatment with JQ1. SUM149-MA and parental SUM149-Luc cells were treated in parallel with 1 or 2 µM of JQ1 for 20 days (treatment killed most of the cells in the parental cell line) and then allowed to recover and grow in a drug-free medium for 5 or 6 days (as indicated) before being stained with crystal violet. Passage numbers: SUM149-Luc, 6 passages in a medium with dialyzed FBS and glutamine; SUM149-MA, 7 passages in a glutamine-free medium followed by 6 passages in a medium with dialyzed FBS and glutamine.

In these experiments comparing JQ1 resistance in SUM149-MA and SUM149-Luc cells, we consistently observed considerably more surviving cells in JQ1-treated SUM149-MA cultures than in SUM149-Luc control cultures. Microscopic images of cells treated with 1 μM of JQ1 for 20 days are shown (Supplementary Figure S1). Figure 1 shows representative images of crystal violet-stained SUM149-Luc and SUM149-MA cells treated with 1 μM or 2 μM of JQ1 for 20 days, followed by 5 or 6 days of recovery in a drug-free medium. Crystal violet staining showed that SUM149-Luc cells treated with 1 μM of JQ1 had only about 10% of the cell mass of SUM149-MA cells with the same treatment. The proportion of surviving SUM149-Luc cells compared to SUM149-MA cells further decreased to about 1% with a JQ1 dose of 2 µM. This result is similar to the results obtained for most agents, including the chemotherapeutic drugs doxorubicin and paclitaxel, demonstrating the highly adaptable and resistant nature of SUM149-MA cells [10,11,12].

### 3.2. Sensitization of SUM149-MA Cells to Chemotherapeutic Drugs by Treatment with JQ1

**Figure 2:**
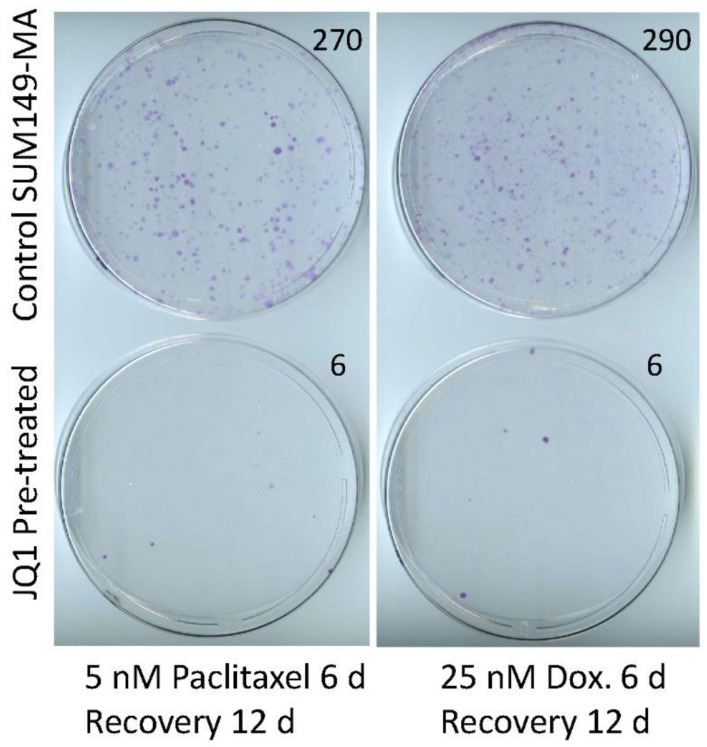
Treatment with JQ1 sensitizes SUM149-MA cells to treatment with chemotherapeutic drugs. After 6 days of treatment with 1 µM of JQ1, we allowed surviving cells to recover in a drug-free medium for 9 days. Following this, we trypsinized the cells, plated them into fresh culture dishes, treated them in parallel with 5 nM of paclitaxel (left panel) or 25 nM of doxorubicin (Dox, right panel) for 6 days, and then let them recover and grow into colonies for 12 days before staining them with crystal violet. Representative images of cell cultures are shown. These images show that pretreatment with JQ1 markedly decreased the number of colonies. Passage number in cell culture: passage 7.

Our main objective was to identify potential therapeutic agents that are noncytotoxic—and therefore suitable for early intervention at the MRD stage—and inhibit resistant cancer cells. Therefore, we investigated whether JQ1 treatment modifies resistant cancer cells toward a nonresistant phenotype. Specifically, we asked whether JQ1 treatment could render resistant cells sensitive to chemotherapeutic drugs, even without direct cell killing. To answer this question, we pretreated SUM149-MA cells with JQ1, allowed them to recover and grow in the absence of JQ1, and then compared their sensitivity to treatment with doxorubicin or paclitaxel with that of control MA cells not pretreated with JQ1. Figure 2 shows the relative resistance of SUM149-MA cells pretreated with 1 µM of JQ1 for 6 days, allowed to recover and grow for 9 days, and treated with 5 nM of paclitaxel or 25 nM of doxorubicin for 6 days (microscopic examination indicated that these treatments eradicated 99% of the cells in all dishes), followed by the recovery and growth of resistant cells into colonies for 12 days. We observed more than 250 colonies of different sizes among the control SUM149-MA cells not pretreated with JQ1. In contrast, SUM149-MA cells pretreated with JQ1 formed markedly fewer colonies after treatment with either paclitaxel or doxorubicin, and we observed only 5–6 colonies per dish (Figure 2). These findings demonstrated that treatment with JQ1, a known modulator of the epigenetic state, rendered the resistant cancer cells sensitive to commonly used chemotherapeutic agents.

### 3.3. Sensitization of SUM149-Luc Cells to Chemotherapeutic Drugs by Treatment with JQ1

**Figure 3:**
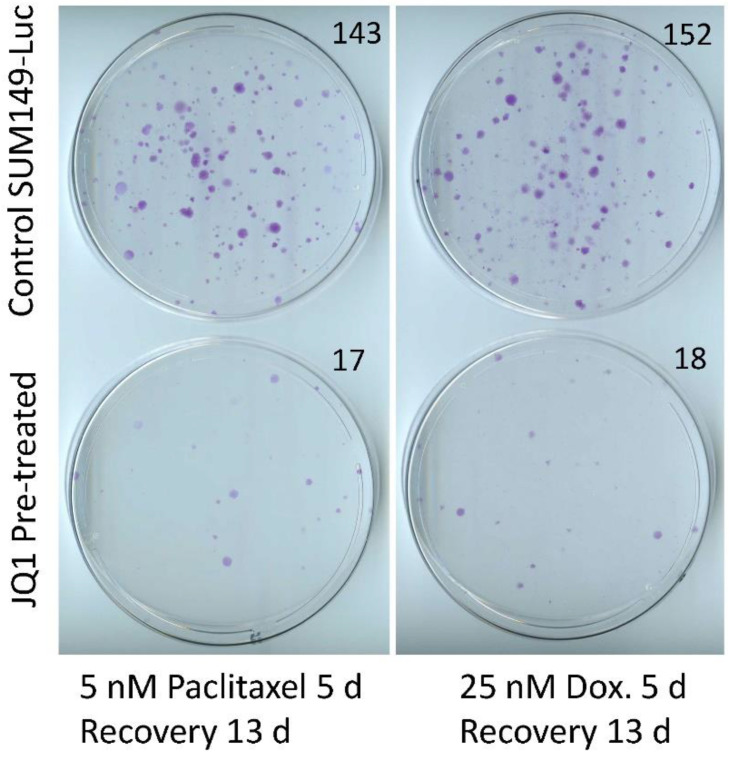
Pretreatment with JQ1 sensitizes SUM149-Luc cells to chemotherapeutic drugs. After 6 days of treatment with 1 µM of JQ1, we allowed the surviving cells to recover in a drug-free medium for 9 days, trypsinized them, and plated them into fresh culture dishes. We then treated the cells with 5 nM of paclitaxel (middle panel) or 25 nM of doxorubicin (Dox, right panel) for 5 days and then let them recover and grow into colonies for 13 days before staining them with crystal violet. These images show that pretreatment with JQ1 markedly decreased the number of colonies. Passage number in cell culture: passage 4.

Although SUM149-MA cells are highly adaptable and resistant, we recognize that the parental cell line SUM149 is one of the most resistant TNBC cell lines. Therefore, we also aimed to determine whether treatment with JQ1 sensitizes resistant cancer cells in the parental SUM149 cell line to chemotherapeutic drugs. To that end, we pretreated SUM149-Luc cells with JQ1 and then tested their sensitivity to paclitaxel and doxorubicin. Specifically, we pretreated the cells with 1 µM of JQ1 for 6 days, allowed them to recover and grow for 9 days, and then treated them with 5 nM of paclitaxel or 25 nM of doxorubicin (5 days of treatment followed by 13 days of recovery for both drugs). Based on the microscopic examination after treatment with chemotherapeutic drugs (prior to recovery), we observed that the treatments eradicated more than 99% of the cells, leaving behind only a few morphologically abnormal cells in total. We found that JQ1-pretreated SUM149-Luc cells formed dramatically fewer colonies than did non-pretreated control cells (15 versus 200 colonies per dish), demonstrating that the pretreatment sensitized SUM149-Luc cells to both chemotherapeutic drugs (Figure 3).

It is noteworthy that we used somewhat different drug treatment and recovery times for SUM149-MA versus SUM149-Luc cells (Figure 2 and Figure 3). We did this to optimize experimental conditions for a particular cell line. SUM149MA cells represent only approximately 0.01% of the subpopulation present in the parental cell line, which survived in quiescence and was reprogrammed to proliferate indefinitely [10,11]; therefore, they behave very differently as compared to the parental cell line. Our main purpose was to determine whether JQ1 pretreatment sensitizes resistant cells.

### 3.4. Combination with JQ1 Enhanced Efficacy of Chemotherapeutic Drugs against Resistant Cancer Cells

**Figure 4:**
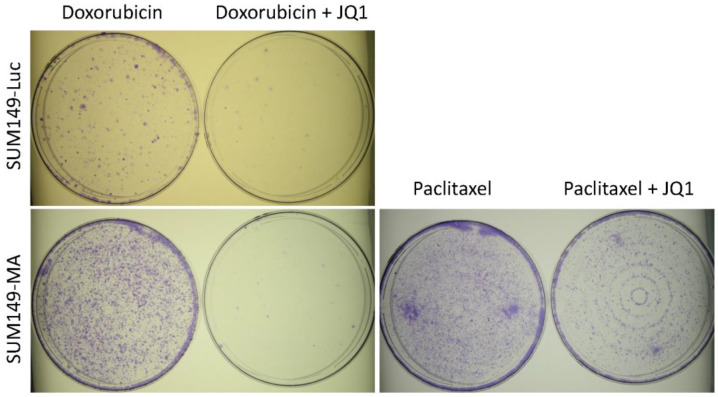
Inhibition of resistant cancer cells treated with a combination of chemotherapeutic drugs and JQ1. SUM149-Luc (top) and SUM149-MA (bottom) cells were treated with 100 nM of doxorubicin or 100 nM of doxorubicin plus 1 µM of JQ1, as indicated (left) for 6 days (which eradicated 99% of the cells in all dishes), and then allowed to recover in a drug-free medium for 11 days before being stained with crystal violet. A comparison of the number of colonies showed that the combination of JQ1 and doxorubicin eradicated more cancer cells in both cell lines than did doxorubicin alone. We also treated SUM149-MA cells with 5 nM of paclitaxel or 5 nM of paclitaxel plus 1 µM of JQ1, as indicated (right) for 6 days, and then allowed to recover in a drug-free medium for 7 days before crystal violet staining. Passage numbers: passage 6 for both cell lines.

In addition to evaluating JQ1 and chemotherapeutic drugs in a sequential manner, we also wanted to determine whether JQ1 treatment would also be effective when used in combination with chemotherapeutic drugs. Combination therapy is commonly used to treat advanced-stage disease when there is an urgency to overcome therapy resistance as soon as possible. We treated both parental SUM149-Luc cells and resistant SUM149-MA cells with 100 nM of doxorubicin alone or in combination with 1 µM of JQ1 for 6 days. Microscopic examination indicated that these treatments eradicated 99% of the cells in all dishes. We then allowed the remaining resistant cells to recover and form colonies in drug-free medium for 11 days and stained them with crystal violet (Figure 4). A comparison of the stained cells showed that doxorubicin-treated SUM149-MA cells formed more colonies than did the parental cell line, as expected. Moreover, the addition of JQ1 along with doxorubicin eradicated substantially more resistant cells in both cell lines than did doxorubicin alone: SUM149-MA cells yielded more than 1000 colonies after doxorubicin treatment, and doxorubicin in combination with JQ1 reduced the number of colonies to fewer than 10 (Figure 4). Although 1 µM of JQ1 alone did not substantially inhibit the growth of SUM149-MA cells after 6 days of treatment (not shown), it sensitized the cells to doxorubicin-mediated cell killing.

We similarly evaluated paclitaxel plus JQ1 combination therapy on resistant SUM149-MA cells. We treated SUM149-MA cells with 5 nM of paclitaxel alone or in combination with 1 µM of JQ1 for 6 days, and then allowed the remaining resistant cells to recover and grow in a drug-free medium for 7 days (Figure 4). These results suggest that the combination of paclitaxel and JQ1 caused more inhibition in SUM149-MA cells than paclitaxel alone. However, the inhibition was modest (approximately 50%) as compared to the experiment where cells were first treated with JQ1 and then with paclitaxel (compare Figure 2 and Figure 4). The likely explanation is that the cytotoxic effects of paclitaxel created an unfavorable environment for JQ1 to optimally reprogram the epigenome in SUM149-MA cells, which may be needed for sensitization.

### 3.5. Decreased PD-L1 Expression in Cancer Cells upon Treatment with JQ1

**Figure 5:**
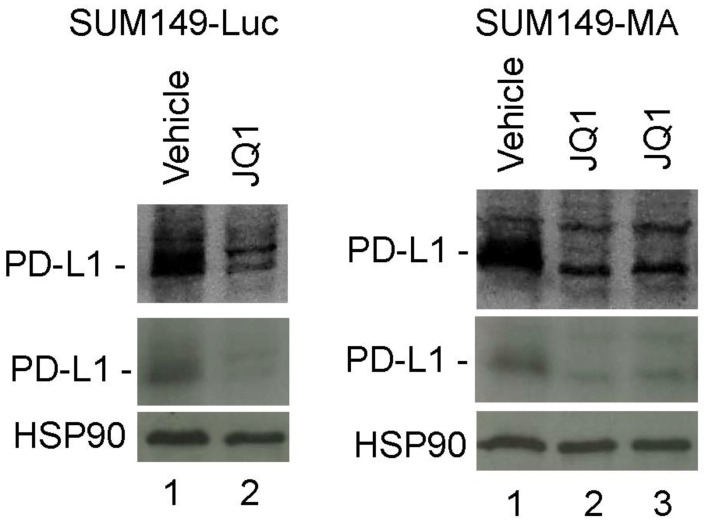
Treatment with JQ1 decreased PD-L1 protein levels in TN-IBC cells. SUM149-Luc (left panel) and SUM149-MA (right panel) cells were treated with 1 µM of JQ1 for 20 days, allowed to recover for 9 days in a drug-free medium (left panel, lane 2; right panel, lane 3), and subjected to Western blotting to compare the PD-L1 protein levels. We also analyzed PD-L1 protein in SUM149-MA cells immediately after 20 days of JQ1 treatment (right panel, lane 2). Nitrocellulose membranes were re-probed with an anti-HSP90 antibody to normalize sample loading. Western blots at the top show darker exposures of PD-L1 blots to better visualize the low-intensity protein bands. Cropped Western blots are shown. See Supplementary Figure S2 for uncropped blots and Supplementary Figure S3 for a similar blot from another experiment. Passage numbers in cell culture: SUM149-Luc, passage 3; SUM149-MA, passage 4.

There is significant clinical interest in immune checkpoint therapy targeting PD-L1 in TNBC. PD-L1 protein on the surface of tumor cells contributes to immune suppression, which promotes tumor progression. It has been shown that JQ1 treatment reduces the expression of PD-L1 in both cancer cells and immune cells (dendritic cells and macrophages), thereby inhibiting cancer progression [25,26]. Here, we investigated whether treatment with JQ1 decreased PD-L1 expression in SUM149-MA cells as part of the reprogramming of the epigenome toward a therapy-sensitive state in TNBC. Western blot analyses showed that 20-day treatment of SUM149-MA cells with 1 µM of JQ1 (followed by a 9-day recovery period) substantially decreased the level of PD-L1 protein (to about 60% that of untreated cells based on a comparison of band intensities; Figure 5, right panel, lane 3). A similar treatment of parental SUM149-Luc cells with JQ1 also substantially decreased the PD-L1 protein levels (to about 50% that of untreated cells; Figure 5, left panel). Since SUM149-MA cells were not as strongly affected by JQ1 treatment as the parental cell line was, we were also able to analyze the PD-L1 level immediately after JQ1 treatment, which showed a similar decrease as observed after a nine-day recovery (compare lanes 2 and 3, Figure 5, right panel). These results suggest that in addition to modulating resistant TN-IBC cells toward a therapy-sensitive state, JQ1 treatment reduced the expression of the immune-suppressive protein PD-L1.

### 3.6. Sensitization of FC-IBC02-MA Cells to Chemotherapeutic Drugs by Treatment with JQ1

**Figure 6:**
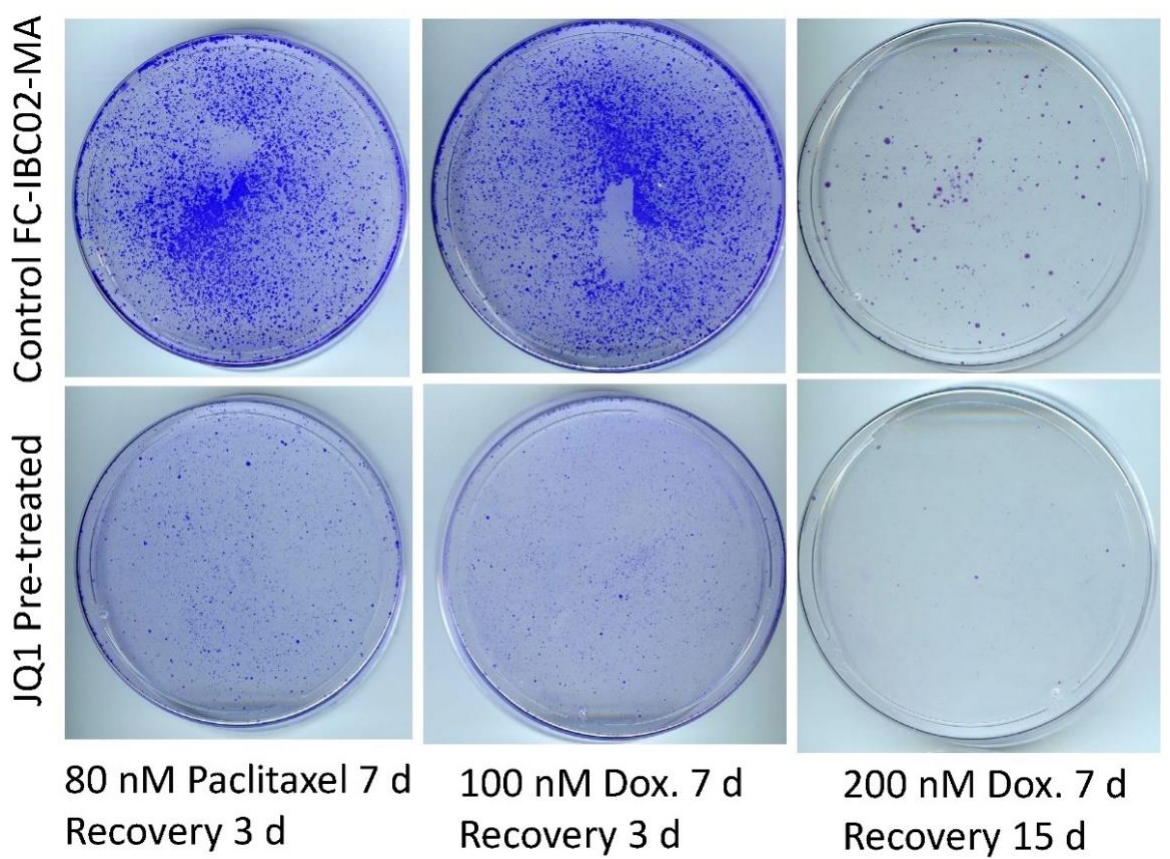
Pretreatment with JQ1 sensitizes FC-IBC02-MA cells to chemotherapeutic drugs. After 6 days of treatment with 62.5 nM of JQ1, we allowed the surviving cells to recover in a drug-free medium for 6 days. We plated the cells into fresh culture dishes and treated with 80 nM of paclitaxel (left panel) or 100 nM of doxorubicin (Dox, middle panel) for 7 days and then let them recover and grow into colonies for 3 days before staining them with crystal violet. We also treated cells with a high dose (200 nM) of doxorubicin for 7 days and allowed the surviving cells to recover for 15 days before staining the colonies (right panel). These images show that pretreatment with JQ1 markedly decreased the number of colonies. Passage numbers in cell culture: FC-IBC02-MA, passage 27; FC-IBC02, parental cell line at passage 21 shifted to glutamine-free medium for 6 passages.

To test whether low-dose JQ1 would affect resistant cells modeled from other TN-IBC cell lines, we used highly chemo-resistant FC-IBC02-MA cells that had been selected in a similar manner as SUM149-MA cells [10,14]. Evaluating a range of drug concentrations, we first observed that FC-IBC02 and FC-IBC02-MA cells were significantly more sensitive to JQ1 treatment than SUM149 and SUM149-MA cells. We chose a relatively low dose (62.5 nM) of JQ1 to investigate whether it would sensitize resistant cells to chemotherapeutic drugs. This dose appeared to be relatively non-cytotoxic since a treatment for six days caused only a modest growth inhibition (less than 50%) and JQ1-treated cells quickly recovered to healthy proliferating cells upon shifting to the medium without JQ1 (Supplementary Figure S4). We found that pretreatment with 62.5 nM of JQ1 sensitized FC-IBC02-MA cells to the chemotherapeutic drugs paclitaxel and doxorubicin. Compared to the control cells, JQ1-pretreated cells yielded a dramatically lower number of cells/colonies (10–20% that of the control) after treatment with chemotherapeutic drugs (Figure 6).

## 4. Discussion

A common concern about any in vitro system, including our cell culture model, is that it is devoid of the tumor microenvironment present in the body, particularly the immune system. In this era of immunotherapy, we consider this issue very important and have approached it in several ways. First, we considered the mechanisms that render immune checkpoint blockade ineffective in cancers where such therapies do not work. There is overwhelming evidence in the literature demonstrating that cancer cells with deep intrinsic resistance, such as that modeled in our system, have several adaptive traits, such as high EMT, that enable them to evade immunity during progression and at the time immune checkpoint therapies are typically offered [30,31]. A recent study has shown that quiescent cancer cells play an important role in resisting T-cell attacks in TNBC [32]. Therefore, a good way to improve the response to immune therapies would be through therapeutic targeting of deep intrinsic resistance, and a cell culture approach such as ours could possibly help in this task.

Second, an ideal way to mitigate the limitation of the missing immune system in a cell culture model is to complement this approach by limiting the evaluation of potential therapeutic agents to those that have desirable characteristics in animal models and humans. For example, there is a tremendous amount of information available from the clinical use of therapies in autoimmune diseases and from past clinical trials in cancer that may point to noncytotoxic compounds that do not harm the immune system and/or modulate it toward a healthier state. If such agents inhibit deep intrinsic resistance in our model, this information, considered in the context of all relevant information from animal models and humans, may justify clinical trials involving combinations of immune checkpoint blockade and other agents for halting/delaying relapse in high-risk breast cancers such as IBC and TNBC. As a good example of this strategy, we have recently reported that the ribonucleoside analog 6-mercaptopurine, which is extensively used for treating autoimmune diseases, inhibited resistant breast cancer cells in our model system [14,15]. Specifically, in choosing JQ1 for this study, we also considered positive results from various studies in animal models and clinical trials showing its efficacy as a single agent and in combination with various therapeutic agents [16,17,18,19,20,21,22,23,24,25,26]. In summary, we believe it is important to use the unique strengths of in vitro and in vivo approaches in a complementary manner to improve drug discovery.

Another important issue is how to interpret the data obtained for JQ1 in our model in a clinical context, i.e., its potential relevance at the MRD stage prior to relapse. Our results are promising because JQ1 treatment sensitized resistant cancer cells to chemotherapeutic drugs. This type of sensitization observed in vitro implies similar sensitization to chemotherapeutic drugs in vivo, which is supported by the results obtained with JQ1 evaluation in animal models of different cancers [16,17,18,22,23]. Further, the cancer cells that are sensitive to chemotherapeutic drugs may also be susceptible for elimination by other selection pressures in the body, such as those imposed by the immune system. Another promising aspect is that we did not see an emergence of resistant clones after several weeks of treatment with JQ1 in cell culture. For agents such as JQ1 to be considered for clinical evaluation, a more rigorous evaluation may involve evaluation of even lower doses for even longer periods in cell culture to determine whether similar results are obtained.

What does the finding that JQ1 treatment reduced PD-L1 expression in cultured cancer cells mean in a clinical context? A lower PD-L1 level in a tumor implies lower immune suppression, which is desirable. We recognize that the relationship between tumors and the immune system varies according to the stage of the disease: the immune system generally functions well when the tumor is at an early stage, but immunity is suppressed in advanced-stage disease. Perhaps this is why it has not been possible to optimize anti-PD-L1 immune checkpoint blockade by aligning it with PD-L1 expression in tumors [1,33]. At this time, PD-L1 antibody therapy is offered to many patients with TNBC, irrespective of their tumor PD-L1 status. Future studies should address whether a combination of JQ1 and immune checkpoint blockade would inhibit metastasis from SUM149-MA-like cancer cells in an immunocompetent mouse model.

Regarding the mechanism of JQ1’s effect on the PD-L1 protein level, there is evidence that JQ1 inhibits BRD4 binding on the PD-L1 promoter, thus inhibiting its expression [25,26]. However, since we have not investigated the mechanism(s) of JQ1 action in our system, there are several possibilities. Since there is a high degree of heterogeneity among MA cells, JQ1’s effect could be mediated through epigenetic changes in all cells or some specific subpopulations of cells. It is also possible that JQ1 does not directly affect the PD-L1 level, but instead a long 20-day treatment causes a shift in subpopulations with high versus low PD-L1 levels (i.e., the cells surviving this treatment have a pre-existing low PD-L1 level). Our goal was not to investigate the mechanisms of JQ1 action, but to investigate through function-based approaches whether JQ1 has the potential to overcome deep intrinsic resistance in our model. We believe MA cells can serve as a good model for investigating the mechanisms of therapy response versus resistance upon treatment with JQ1 and other clinical-grade bromodomain inhibitors in the context of adaptable cancer cell states in the future.

## 5. Conclusions

Our results obtained in a cell culture model of deep intrinsic resistance, featuring highly adaptable TNBC cells that opportunistically switch between quiescence and proliferation, suggest that the BET bromodomain inhibitor JQ1 may be useful for sensitizing resistant breast cancer cells at the MRD stage in patients with breast cancer at high risk of recurrence. Our results are consistent with the results obtained with JQ1 in other model systems of therapy resistance, representing several different cancers, including breast cancer. These results support the usefulness of modeling a realistic resistance phenotype in cancer by incorporating body-like selection pressures in cell culture, which can be used for evaluating noncytotoxic agents that may be suitable for use before the disease advances to metastasis.

## Data Availability

The data presented in this study are available in the article (and the Supplementary Materials).

## Supplementary Materials

The following are available online at https://www.mdpi.com/article/10.3390/cancers15072036/s1, Figure S1: Resistance of SUM149-MA cells to treatment with JQ1. Figure S2: Uncropped Western blots used for Figure 5. Figure S3: An additional PD-L1 Western blot similar to the one shown in Figure 5. Figure S4: Effect of low-dose JQ1 on the morphology of FC-IBC02 and FC-IBC02-MA cells.

## Abbreviations

DMSO: dimethyl sulfoxide; EMT: epithelial to mesenchymal transition; FBS: fetal bovine serum; IBC: inflammatory breast cancer; MA: metabolically adaptable; MRD: minimal residual disease; TNBC: triple-negative breast cancer; TN-IBC: triple-negative inflammatory breast cancer.
