# Supplementary material for: Sensitization of Resistant Cells with a BET Bromodomain Inhibitor in a Cell Culture Model of Deep Intrinsic Resistance in Breast Cancer
Source: Cancers (Basel). 2023 Mar 29;15(7):2036. doi: 10.3390/cancers15072036 (PMC10093448; doi:10.3390/cancers15072036)
Supplement: Supplementary file 1 [file cancers-15-02036-s001.zip › cancers-2259716-supplementary.pdf]

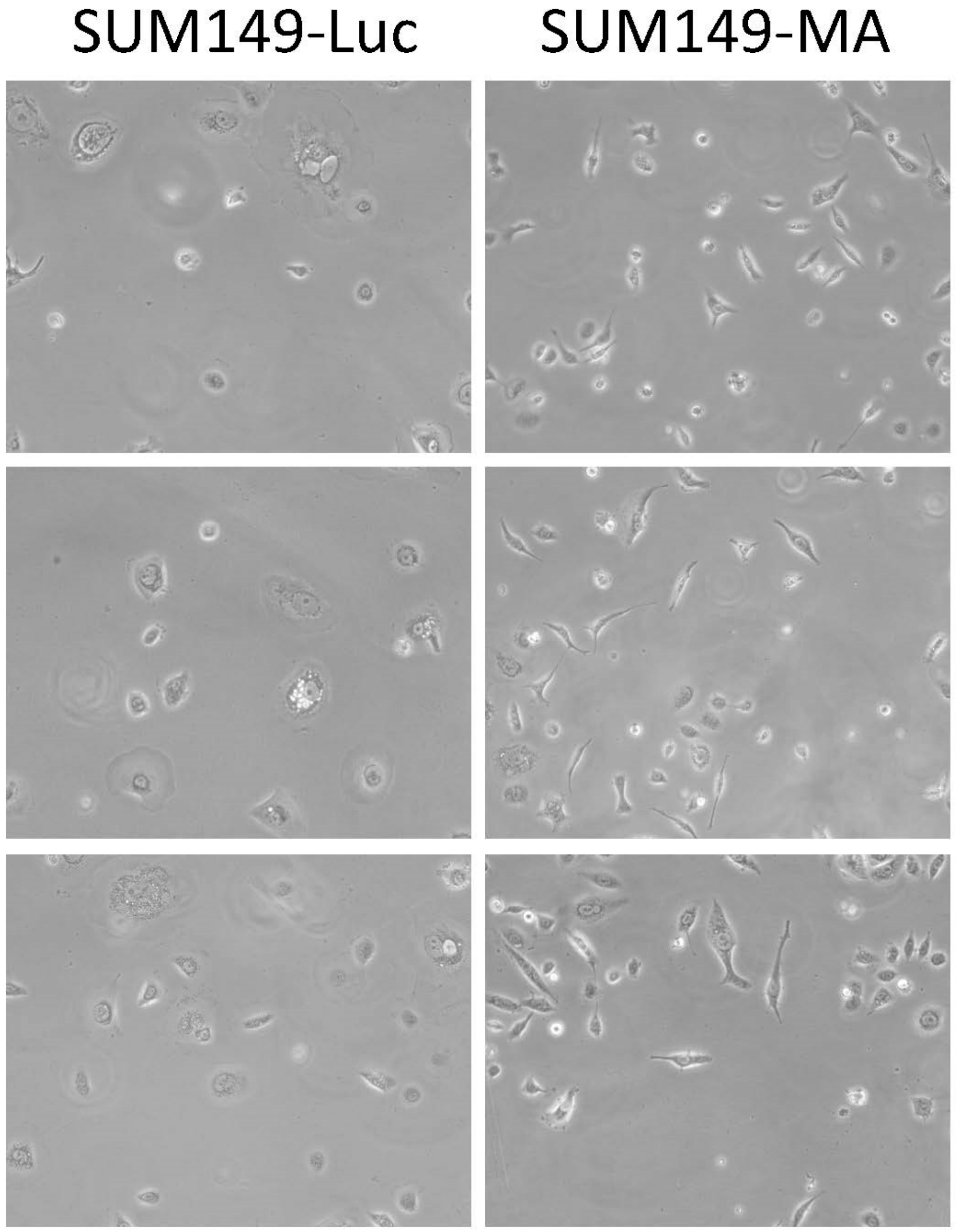

Supplementary Figure S1. Resistance of SUM149-MA cells to treatment with JQ1. We treated SUM149-MA and parental SUM149-Luc cells in parallel with 1 JQ1 for 20 days and then collected representative images under microscope at 10x magnification. Triplicate images are shown. Passage numbers in cell culture: passage 5 for both the cell lines.

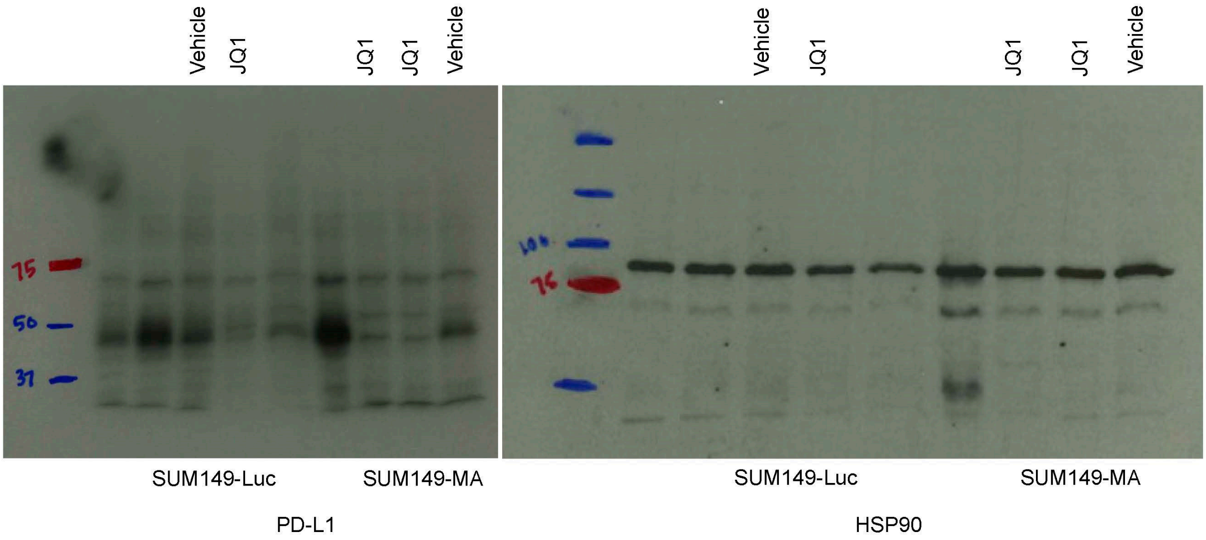

Supplementary Figure S2. Uncropped Western blots used for the cropped blots shown in Figure 5. The lanes used in Figure 5 are labeled, and the cell lines and JQ1-treated lanes are identified. Primary antibodies used for the Western blots are shown at the bottom.

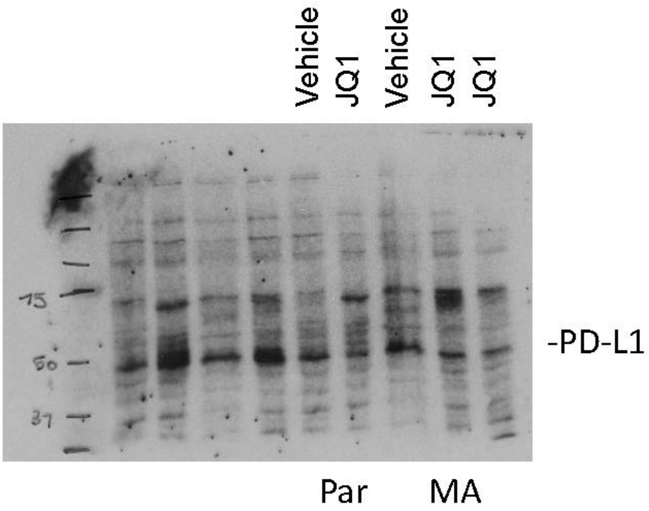

Supplementary Figure S3. Additional PD-L1 Western blot similar to the one shown in Figure 5. The cell lysates were identical to those used for Figure 5. Par: parental SUM149-Luc cell line, MA: SUM149-MA cell line. Two different lysates from JQ1-treated MA cells were prepared after 20 days treatment followed by a 9 days recovery (lane next to control vehicle treated lane), or after 20 days treatment (no recovery; rightmost lane).

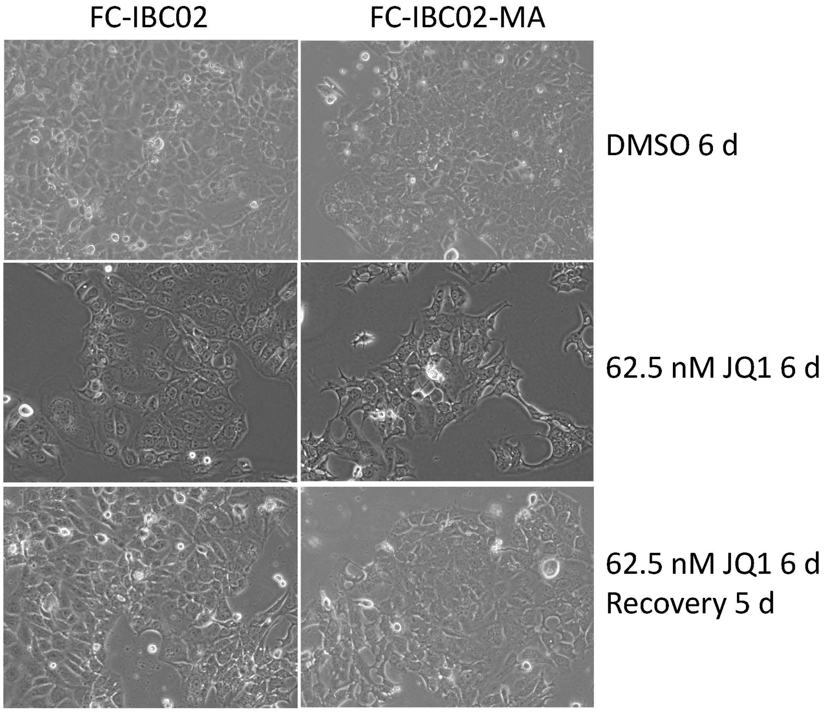

Supplementary Figure S4. Effect of low-dose JQ1 on morphology of FC-IBC02 and FC-IBC02-MA cells. We treated cells with 62.5 nM JQ1 (middle panel) or solvent DMSO (upper panel) for 6 days and then collected representative images under microscope at 10x magnification. We switched the JQ1-treated cells to the medium without JQ1 for 5 days, and then photographed (lower panel). Passage numbers in cell culture: FC-IBC02, passage 26; FC-IB02-MA, parental cell line at passage 21 shifted to glutamine-free medium for 4 passages.
